# Sensory collectives in natural systems

**DOI:** 10.7554/eLife.88028

**Published:** 2023-11-29

**Authors:** Hannah J Williams, Vivek H Sridhar, Edward Hurme, Gabriella EC Gall, Natalia Borrego, Genevieve E Finerty, Iain D Couzin, C Giovanni Galizia, Nathaniel J Dominy, Hannah M Rowland, Mark E Hauber, James P Higham, Ariana Strandburg-Peshkin, Amanda D Melin

**Affiliations:** 1 Department of Migration, Max Planck Institute of Animal Behavior Konstanz Germany; 2 Centre for the Advanced Study of Collective Behaviour, University of Konstanz Konstanz Germany; 3 Department of Biology, University of Konstanz Konstanz Germany; 4 Department for the Ecology of Animal Societies, Max Planck Institute of Animal Behavior Konstanz Germany; 5 Zukunftskolleg, University of Konstanz Konstanz Germany; 6 Department of Collective Behaviour, Max Planck Institute of Animal Behavior Konstanz Germany; 7 Department of Anthropology, Dartmouth College Hanover United States; 8 Max Planck Research Group Predators and Toxic Prey, Max Planck Institute for Chemical Ecology Jena Germany; 9 Advanced Science Research Center and Program in Psychology, Graduate Center of the City University of New York New York United States; 10 Department of Anthropology, New York University New York United States; 11 Department of Anthropology and Archaeology, University of Calgary Calgary Canada; 12 Alberta Children’s Hospital Research Institute, University of Calgary Calgary Canada; University of Zurich Switzerland; University of Zurich Switzerland

**Keywords:** umwelt, perception, collective movement, sensory ecology, animal behaviour, complex systems

## Abstract

Groups of animals inhabit vastly different sensory worlds, or umwelten, which shape fundamental aspects of their behaviour. Yet the sensory ecology of species is rarely incorporated into the emerging field of collective behaviour, which studies the movements, population-level behaviours, and emergent properties of animal groups. Here, we review the contributions of sensory ecology and collective behaviour to understanding how animals move and interact within the context of their social and physical environments. Our goal is to advance and bridge these two areas of inquiry and highlight the potential for their creative integration. To achieve this goal, we organise our review around the following themes: (1) identifying the promise of integrating collective behaviour and sensory ecology; (2) defining and exploring the concept of a ‘sensory collective’; (3) considering the potential for sensory collectives to shape the evolution of sensory systems; (4) exploring examples from diverse taxa to illustrate neural circuits involved in sensing and collective behaviour; and (5) suggesting the need for creative conceptual and methodological advances to quantify ‘sensescapes’. In the final section, (6) applications to biological conservation, we argue that these topics are timely, given the ongoing anthropogenic changes to sensory stimuli (e.g. via light, sound, and chemical pollution) which are anticipated to impact animal collectives and group-level behaviour and, in turn, ecosystem composition and function. Our synthesis seeks to provide a forward-looking perspective on how sensory ecologists and collective behaviourists can both learn from and inspire one another to advance our understanding of animal behaviour, ecology, adaptation, and evolution.

## Introduction

Collective behaviour produces some of the most captivating phenomena on Earth, from the swarming of billions of locusts (Ariel and Ayali, 2015; Romanczuk et al., 2009) and coordinated movements of fish schools (Georgopoulou et al., 2022), to the construction of complex architecture (Laidre, 2021; Reid et al., 2015) and coordination of cooperative breeding (Shen et al., 2017). Research on collective behaviour seeks a predictive and analytical understanding of the coordination of behaviours among individuals. To coordinate behaviour, animals use their sensory systems to acquire and process signals and cues from the environment, and from other individuals. How information is gathered and processed, and how it contributes to the collective behavioural decisions and outcomes are, however, seldom integrated into studies of collective behaviour. Such integration would help ensure that analytical models consider the perceptual and cognitive reality of the focal animal (Bastien and Romanczuk, 2020; Rosenthal et al., 2015). In turn, stimuli generated by collectives are likely to exert selective pressures back on sensory systems, but likewise, this has rarely been considered by sensory ecologists. By examining these dynamics, we can better understand the morphology, physiology, behaviour, and evolution of species and ecosystems, and how changing environments might impact their survival and conservation.

In this review, we advocate that integrating sensory ecology into collective behaviour will expand conceptual and methodological approaches and bring new depth and discovery to the field of collective behaviour. We start by describing the current status of the two fields and set forth our case for their further integration. We then introduce the idea of the sensory collective, where different sensory inputs are perceived and shared via signals or cues among members of a group and integrated into an overall collective perception. In doing so, we discuss the potential benefits afforded by the increased sampling range of the collective and discuss the potential costs of group-induced noise. We then consider how the study of collective behaviour may provide new insights and models for understanding the evolution of sensory systems, assessing whether selective pressures are exerted by sensory collectives. Such an approach has yet to be thoroughly investigated in sensory ecology, but is likely to generate new insights into forces shaping the form and functions of visual, olfactory, haptic, thermal, auditory, magnetic, gustatory, and other sensory systems. With this goal in mind, we highlight a few examples of systems in which further study of how collective stimuli might shape the evolution of sensory systems seems likely to be fruitful. Building on this, we offer some examples of how collective stimuli might shape the evolution of sensory systems and discuss neural circuits involved in sensing and collective behaviour. We illustrate this by focusing on examples from diverse taxa and highlighting the promise of shared model systems in furthering this goal. Importantly, combining collective behaviour and sensory ecology requires new methods and theoretical approaches. To this end, we review emerging conceptual and methodological advances, and identify testable hypotheses and potential applications suitable for driving this research forward towards quantifying a ‘sensescape’. We explore what it will take to understand the combined and intersecting sensory perceptions of social individuals and, more critically, how sensory systems scale up to the level of group decision-making and the resulting collective behaviour. We conclude by discussing the applications and significance of sensory collectives for biological conservation**,** arguing that determining the umwelten of a sensory collective is essential for understanding how animals respond to sensory pollution and anthropogenic change. Given the increasing impact of human populations on animal ecology and population dynamics, we suggest understanding the sensory ecology of collectives will offer important insights into conservation initiatives.

## Integrating collective behaviour and sensory ecology

Research in collective behaviour has begun to explore the sensory capabilities of organisms, and the relevant signals and cues that may be perceived and influence collective behavioural decisions. Such efforts have yielded important insights, including the complex nature of behavioural contagion and information transfer in fish schools (Strandburg-Peshkin et al., 2013), the trade-offs between efficient foraging and conspecific interference in echolocating bats (Cvikel et al., 2015), and the use of specific calls in maintaining cohesion in meerkat groups (Gall and Manser, 2017). Most research programs on collective behaviour, however, do not yet ground the research in the concept of umwelten (singular, umwelt; i.e. the world as it is experienced by a particular organism, reviewed in Caves et al., 2019; Partan and Marler, 2002), nor do they consider the sensory constraints of different species (Lemasson et al., 2009; Pita et al., 2016). This is where sensory ecology has much to offer to the study of collective behaviour.

The goal of sensory ecology is to understand how organisms perceive and interact with their social and physical environment, and in turn how the environment shapes the form and function of sensory systems (e.g. Cuthill et al., 2017). In recent decades many advances in the study of animal sensory ecology have emerged, including the application of sophisticated models of animal vision (Cassey et al., 2008; Olsson et al., 2018; Stöckl and Kelber, 2019; Troscianko and Stevens, 2015; Vorobyev and Osorio, 1998), methods to investigate chemotaxis and olfactory assessment (Jacobs, 2012; Nevitt, 2008), and the use of comparative genetics to understand the proteins underlying mechanosensation (Aiello et al., 2018; Bagriantsev et al., 2014) and electro signal selection during electroreception (Crampton, 2019; Montgomery et al., 2014). Sensory ecology has also been driving forward the conceptual framework for studying multimodal perception (Dominy et al., 2016; Higham and Hebets, 2013; Melin et al., 2022; Stynoski and Noble, 2012) highly relevant to collective behaviour; where the repeated interactions among many individuals not only produce patterns on a scale much larger than the individuals themselves, but involve integrating signals and cues at different scales involving combinations of sensory modalities, such as vision, sound, chemosensation, and electric and magnetic fields (Sumpter, 2010).

Because the emergent properties of these associations underlie diverse aspects of daily life for many species, they also have consequences for behavioural plasticity (Jordan and Ryan, 2015). Collective behaviour is vital not only for understanding the sociality, ecology, and evolution of animals, but also for estimating resilience of species to environmental variation; a pressing concern in light of pervasive climate change (Berdahl et al., 2016; Hung et al., 2014; Hurme et al., 2022). Accordingly, efforts to improve and enhance the toolkit for investigating collective behaviour are of high importance for biological conservation (Westley et al., 2018).

**Figure 1.**
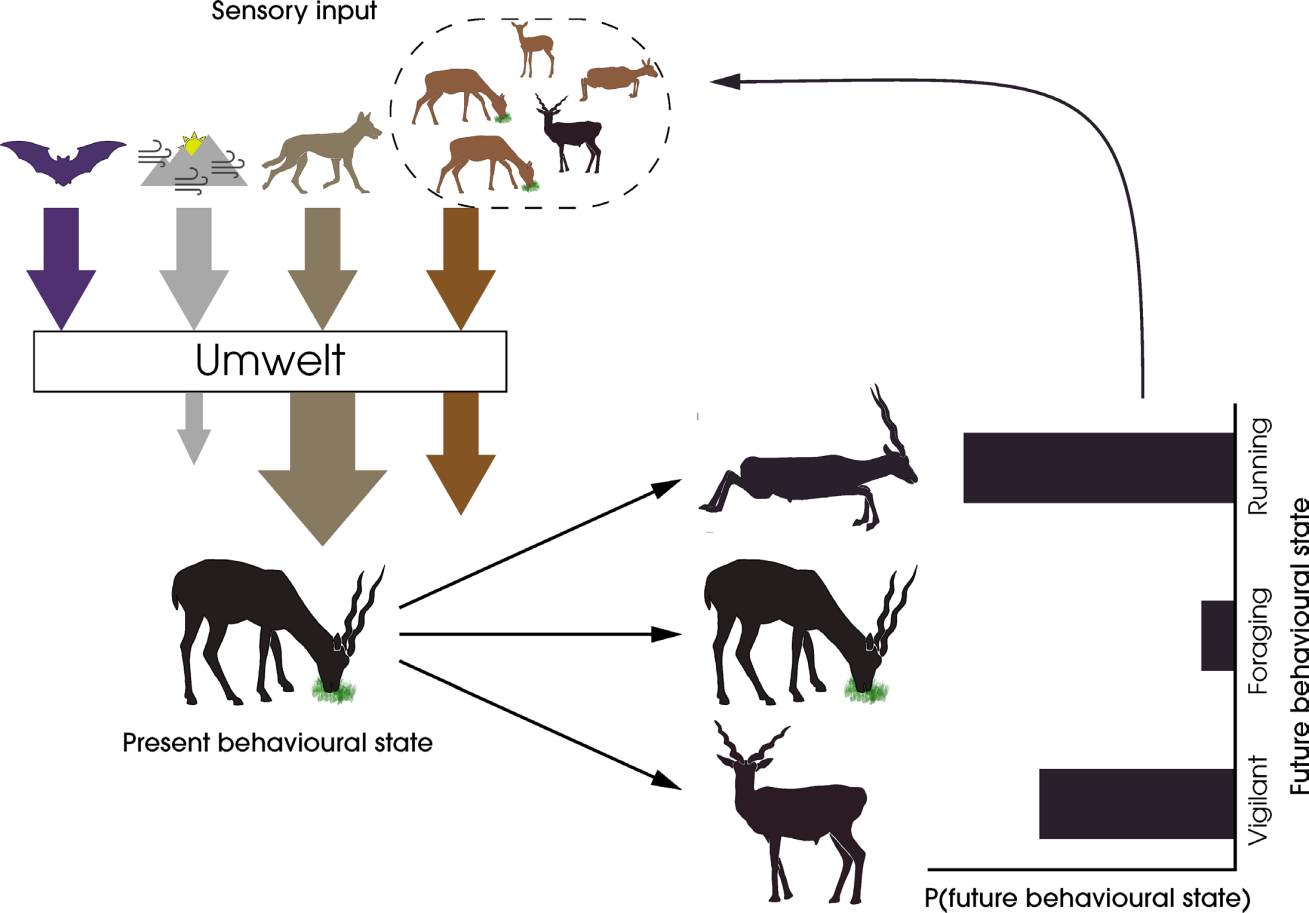
A framework for the study of sensory collectives. This schematic highlights the feedback between the sensory stimuli experienced by animals and their behavioural states. The arrows represent the different types of stimuli experienced by the prey animal—harmless and potentially harmful heterospecifics (purple and beige, respectively), social conspecifics (orange), and the physical environment (grey). The animal’s selective perception of the stimuli by filtering, amplification, or reduction creates an umwelt, the response to which determines the animal’s future behavioural state. In this specific example, the sensory system of the foraging blackbuck prevents the detection of echolocation calls of a bat, amplifies predator cues from a wolf, filters out cues from the physical environment, and attends fully to conspecific cues to form its personal umwelt. The integration of these cues leads to a probabilistic change in the behaviour of the individual, which in turn feeds back into the sensory systems of other conspecifics.

Integrating sensory ecology with collective behaviour has the potential to establish the umwelt of a species, which is essential for understanding which stimuli are perceived, filtered, prioritised, and integrated downstream in both individual and collective decision-making processes. In Figure 1, we consider the umwelt of a blackbuck (*Antilope cervicapra*) experiencing a predatory threat. Here, out of the multiple sources of high-dimensional sensory inputs, the sensory and attentional systems of the animal filter out irrelevant input, amplifying the salience of the predatory threat, both directly and indirectly via conspecifics, and reducing cumulative stimuli from the physical environment. As a result of this filtering, the animal lives in a personal umwelt, influenced by the group. Recognising such nonlinear transformations of the sensory input available to individuals is essential for quantifying animal responses to their environment and, in doing so, predicting their future behaviours. The answers to pressing questions in the study of collective behaviour might be found through creative integration of sensory ecology research (Box 1).

Box 1. **Questions to advance the study of collective behaviour integrating a sensory perspective**

- How can we incorporate the umwelten of group-living actors to predict behavioural outputs and emergent collective properties (Figure 1)?
- How is the sensory perception of a collective correlated among individuals?
- How do individuals within a collective differ in the perception of potentially informative signals and cues, and how does downstream feedback influence their behaviour?
- How can we quantify environmental stimuli and perception by individuals to better understand individual and group responses to stimuli?
- What are the individual computational costs and benefits of an efficient sensory collective?
- How has the anthropocene impacted sensescapes, sensory abilities, and emergent properties of sensory collectives of species, especially those most vulnerable to change?

## The concept of ‘a sensory collective’

Sensory systems and downstream cognitive pathways integrate inputs across multiple receptor types, sensory modalities, and brain regions, with processes such as summation, inhibition, and opponency determining the resultant perception (Bogacz, 2007). We might envisage a sensory collective as a system where the members receive different sensory inputs, which are then shared via signals and cues, and integrated into an overall collective perception. This then may trigger a group-level response and inform collective decision-making (Arganda et al., 2012). With discrete and overlapping individual umwelten, the emergent property of a sensory collective may be in the power of increased dimensionality for an appropriate behavioural response. While the total dimensionality of the system would be cognitively expensive for any individual to process within a group, the pooling of information (Webster et al., 2017) from a group of individual umwelten would suggest that the per capita cognitive cost may be reduced. This pooling of information in groups could function analogously to the skill pool effect of social foraging, whereby individuals in a group benefit from diverse foraging specialisations (Giraldeau, 1984), but in this case the benefit would derive from sensory specialisation. Yet, the processing required to integrate umwelten within the collective and its influence on the group behaviour is little understood (but see applications of Bayesian approaches at the individual and collective level; Arganda et al., 2012; Cheng et al., 2007).

As the number of sensory receptors in the system increases, the possibility of noise arising from processing the inputs arriving from multiple receptors increases, as does the probability of corrupting the signal or an un-coordinated response. This may arise even if all individuals within a collective are aligned in their interests over the outcome of the signalling interaction, such as when navigating to a food patch when group foraging, or, for example, when individuals in a cohesive group align with others in a self-organised escape response to a predator (Papadopoulou et al., 2022). This becomes further complicated in scenarios where individuals of the collective have different interests (such as in decisions over mate choice and reproduction). The task of attempting to integrate many different umwelten, motivations and internal states (i.e., physiological) is a recipe for a noisy informational state and could lead to a scenario where the wisdom of the crowd is much less than the wisdom of its constituents (Laland and Williams, 1998). Such group-induced noise (noise that is created by the presence of multiple signallers who give different signals concurrently) is likely to be modulated by group size and composition. For example, the proportion of naive individuals impacts the number of ‘mistaken’ or mismatched reactions to stimuli and also the time to reach a behavioural consensus (Couzin et al., 2005; Couzin et al., 2011), but in small group sizes (or effective group sizes given group hierarchies and composition, see below) some noise in the system can enhance decision accuracy as it removes the constraints of highly correlated information, particularly in complex environments (Kao and Couzin, 2014; Box 2). Game theoretic models of multiple signallers show that information can be shared cheaply and reliably when there is alignment of interests between the communicating individuals (e.g. Crawford and Sobel, 1982), for example, as in the case of a flocking group of birds avoiding a predator. However, under circumstances in which individuals either have conflicts of interests or have interests that are only in partial alignment, the situation is more complicated. Nonetheless, individuals may receive fully revealing information both cheaply and reliably when they have alignment of interests over the outcome with at least some of the signallers. The key to this is in the ability of individuals to pay attention to the signals of those with whom they share interest alignment on each particular issue, while discounting the signals of those who they do not (Wilson et al., 2013).

Box 2. **A mechanism for an adaptive benefit of sensory collectives**

Decision-making is based on imperfect prior expectations, but an individual may decrease uncertainty in this expectation by incorporating social cues as a source of up-to-date information. If we consider efficiency as reduced energetic expenditure (Williams and Safi, 2021), the adaptive value of the sensory collective can be quantified in terms of the energetic efficiency of the behaviour of its individuals, which should be greater than can be achieved if acting (sensing) independently. Numerous inputs or the introduction of noise in the system by the collective would decrease the overall probability of an incorrect decision in small groups and more so in unpredictable or highly dynamic environments (Kao and Couzin, 2014). The collective decision accuracy is therefore influenced by the group size and the reliability of a cue given environmental dynamism or predictability. Exploring the effect of group size and composition on energetic efficiency of behaviour outcomes under different environmental contexts will reveal the adaptive value of collective sensing. For example, there will likely be critical thresholds such as the number of individuals needed to reduce uncertainty in decision-making for a group to migrate through a highly dynamic environment. Such a threshold would likely differ to that required to find a food patch on which to forage if this resource is highly predictable in time and space. Understanding how the energetic value of the collective differs according to uncertainty in decision-making and environmental complexity could shed light on phenomena such as flexible and variable patterns of association and animal movement.

The diversity of sensory inputs perceived by multiple individuals, when combined with an ability for communication and multi-modal signalling among group members, can lead to increased sampling of the environment as an emergent property of a collective. In this scenario, individuals benefit from reduced uncertainty such that a more appropriate response to the environment can be achieved than if reliant on personal information alone. The propagation of sensory information through a collective may effectively increase the perception range for all individuals in the group, allowing individuals to sense what they are not directly exposed to, that is, the Trafalgar effect (Treherne and Foster, 1981). For example, echolocating bats can adjust their movements with conspecifics while foraging, creating mobile sensory networks to better eavesdrop on neighbours encountering prey, thus extending their perceptual range far beyond that of their individual echolocation detection (Roeleke et al., 2022). Similarly, visual cues and signals are used by many animal groups across taxa to quickly respond to predator threats (Davidson et al., 2021; Kastberger et al., 2008; Pita et al., 2016). For example, as seen in golden shiner fish (*Notemigonus crysoleucas*), visual cues from conspecifics underlie information transmission during collective responses to perceived predation threats (Rosenthal et al., 2015). The benefit is a global response to perturbations experienced by a few (or many) individuals that can subsequently propagate through the group (Cavagna et al., 2010; Kastberger et al., 2008). With an increased effective perceptual range, the collective is also able to detect large-scale environmental gradients, while filtering noise at relatively smaller spatial scales (Berdahl et al., 2013).

The social propagation and informative potential of stimuli may be constrained by the stimuli modalities, and by properties of the sensory collective, namely the physical properties of the collective and the environment. For example, acoustic, chemical (olfactory and gustatory), haptic, electric, and visual signals and cues have different active spaces within a given environment with respect to their persistence in time and spatial attenuation (Brenowitz, 1982; Davidson et al., 2021; Patricelli et al., 2007; Stanley et al., 2016; Veilleux et al., 2022). Turning to the sensory collective, the properties of the spatial distribution of group members will affect detection relevance of the original stimuli. For example, in densely aggregated groups, the range of visual perception may be constrained if group members are blocking the view (e.g. Davidson et al., 2021). Furthermore, visually influenced contagion effects have been demonstrated in schooling fish (Harpaz et al., 2021; Rosenthal et al., 2015; Sosna et al., 2019; Strandburg-Peshkin et al., 2013) and human crowds. Group contexts will also impact other senses. For example, use of olfaction, which is the primary sense of many insects (Szyszka and Galizia, 2015), will be impacted by upstream group members disrupting odour plumes. Most of the research on sensory perception has been measured in relatively few well-studied systems (schooling fish, with some works exploring sensory occlusion in non-animal robotic and UAV group systems; e.g. Poel et al., 2021; Schilling et al., 2022) and/or under controlled laboratory conditions, prohibiting an understanding of taxonomic variation and responses under natural contexts. Future research could usefully expand the species studied to enable cross-species comparisons to be made. We note that some examples with terrestrial groups predict an effect of sensory occlusion in group behaviour such as hunting (Hansen et al., 2023; Lima, 1995), but that creative experiments are required to assess social-dynamic impacts on sensory cue and signal perception quantifiably in these systems. Importantly, such studies should recognise that while individuals might perceive some information with one modality (e.g. visually) they may transmit this information through other modalities to their conspecifics (e.g. through acoustic or haptic signals and cues) (Arnold et al., 2008; León et al., 2022). More generally, further integration of empirical and modelling work (Harpaz et al., 2021) is needed to understand how the sensory capacities of different species shape their groups’ spatial structure and collective responses to the environment, and vice versa.

The differentiation of ‘roles’ of individuals, for example, presence of designated ‘sentinels’ as seen in meerkats (*Suricata suricatta*), also impacts signalling properties and processes, and the correlation of information between group members. There may also be an optimal density or structure of the collective shaped by the spatio-temporal properties of the environment. For instance, in moving groups within temporally dynamic environments, the sensory collective increases certainty of the future potential state for any individual (Williams and Safi, 2021). However, the value of information depends on the dynamism of the environment, for example, when soaring animals access locations of thermal updrafts by observing conspecifics, large spatial separation between individuals may make this information obsolete by the time they can make use of it. We can hypothesise that individuals maximise access to information at the most relevant spatio-temporal scales by adjusting cohesion within the group through (i) minimising information redundancy from overlapping sensory ranges or (ii) maximising information relevancy by optimising inter-individual separation. Fission–fusion societies—where group composition changes over time—may provide an interesting opportunity for studying the costs and benefits of membership in groups with different properties, while allowing some ability to control for variation driven by individuals and environments.

Emergent collective behaviours can create additional sensory cues (Figure 1), which in turn may further inform subsequent actions and exert new selective pressure on sensory collectives. For example, a flock of storks moving in tight cohesion to exploit a thermal updraft essentially ‘maps’ the structure of the thermal (Nagy et al., 2018) providing a visual cue of updraft availability to outside observers. In this example, collective movement shaped the sensescape of individuals within and beyond the collective. Similarly, due to the sheer number of starlings in a murmuration (Young et al., 2013), waves of directional changes produce an emergent auditory cue that may propagate more effectively than visual stimuli (Papadopoulou et al., 2022), reinforcing the higher level emergent cue. In bees, the build-up of alarm pheromone from a few bees increases attractiveness, creating a better response; however, when even more bees release alarm pheromone, the effect is repellent—thus the collective chemical communication regulates the number of bees involved in defence (Petrov et al., 2022). These ideas invite future testing; increasing our understanding of the propagation of physical and social stimuli and behaviours will allow more refined approaches and promote understanding of emergent collective properties and their evolutionary significance. In turn, properties of social collectives are anticipated to shape the evolution of animal sensory systems.

## The evolution of sensory systems: Are selective pressures exerted by sensory collectives?

Recent decades have witnessed huge advances in our understanding of the selective pressures shaping sensory system evolution in a large range of organs and organisms (Melin et al., 2022; Moore et al., 2017; Oteiza and Baldwin, 2021; Zhao et al., 2009). Many research programs have focused on ecologically relevant stimuli and environments such as the impacts of light levels during activity on visual system evolution, including eye shape, size, and photoreceptor composition (Mitra et al., 2022; Moritz et al., 2014; Peichl et al., 2000; Veilleux and Kirk, 2014), the organic compounds in potential foods and the types, structure, and distributions of taste receptors (Baldwin et al., 2014; Chu et al., 2014; Cramer et al., 2022; Jiang et al., 2012; Toda et al., 2021; Zhao et al., 2015), and prey movements and the localisation and sensitivity of mechanical and electric receptors in predators (Gardiner and Atema, 2007; Niesterok et al., 2017; Pohlmann et al., 2004; Schulte-Pelkum et al., 2007). Research into social factors and their impact on sensory evolution has also generated important insights. For example, olfactory receptors underlying the sense of smell are well tuned to volatile organic compounds excreted in body and urinary odours, facilitating complex behaviours ranging from mother–offspring bonding to mate attraction and territorial defence (Ache and Young, 2005; Lübke and Pause, 2015; Wyatt, 2014). Pheromones produced by social insects are also well studied and have contributed to our understanding of insect societies and ecological impacts (Sharma et al., 2015; Yan et al., 2017). For example, a species-specific queen pheromone in several hymenopteran species suppresses workers from reproducing, thus maintaining reproductive division of labour (Van Oystaeyen et al., 2014). The study of olfactory pheromones is particularly interesting because both the biosynthesis of the pheromone in dedicated glands and the sensory detection of the pheromones via selective olfactory receptors have evolved together. Interestingly, this evolution happens within an umwelt that is different for each species: often, animals are anosmic to pheromones from distant species, which means that pheromone communication is often private within a species (Galizia, 2014; Galizia, 2008). The colour of sexual skin is also proposed to exert selective pressures on colour vision evolution of primates, birds, lizards, fish, and many other animals (Caro and Mallarino, 2020; Cooney et al., 2019; Dunn et al., 2015; Fernandez and Morris, 2007; Hiramatsu et al., 2017; Maruska and Butler, 2021; Moreira et al., 2019; Stoddard and Hauber, 2017).

We know far less—and have seldom asked—how the nature of cues generated by collectives of conspecifics may shape sensory system evolution. Such as, how does the grouping behaviour of social primates facilitate or hinder the detection and selection of skin signals? How does aggregation of many individuals of carnivore species into the same den influence olfactory assessment of potential mates? Important exceptions to the rule further demonstrate the promise of such an approach. For example, ornithologists, mammalogists, and entomologists have asked how parents find and identify their own offspring in massive breeding colonies. Their results point to a keen ability for individual recognition via odour (Caspers et al., 2013; Leedale et al., 2021; Liang et al., 2021; Loughry and McCracken, 1991), vocalisations (Knörnschild et al., 2020; Lefevre et al., 1998; Lengagne et al., 2001; Wilkinson, 2003), or colour/pattern (Quach et al., 2021; Stoddard et al., 2014; Tibbetts and Dale, 2007). The selective pressures of discriminating among hundreds to thousands of relatively similar stimuli generate testable hypotheses about the mechanisms and limitations of sensory tuning. These pressures might impose constraints on the evolvability of other sensory dimensions. For example, a trade-off between olfactory sensitivity to a small number of similar odorants versus the ability to detect a breadth of odorant types might occur as the number of olfactory neurons, and the size of cognitive regions devoted to olfactory processing in the brain, are likely limiting factors (Healy and Guilford, 1990). Similarly, pheromones and their dedicated olfactory receptors coevolve within a species (see above). A better understanding of the types of sensory stimuli generated in collective contexts, and their relative importance, will add to comprehensive frameworks for studying sensory system evolution.

## Neural circuits involved in sensing and collective behaviour: Examples from diverse taxa

Collective behaviour in groups of animals typically arises from the detection and response to signals or cues from conspecifics. While recent developments in neurobiology have suggested a shared vertebrate forebrain circuitry involved in social decision-making (O’Connell and Hofmann, 2012) and, in particular, the existence of social place cells (neurons that encode the position of and observed conspecific) in the mammalian brain (Danjo et al., 2018; Omer et al., 2018), little is known about neural circuits that are involved in orchestrating collective behaviours in larger and more complex groups. With this goal in mind, we highlight a few (far from exhaustive) examples of systems in which further study of how collective stimuli might shape the evolution of sensory systems seems likely to be fruitful.

Birds reproduce and feed in a wide diversity of social and ecological contexts, and ornithology offers numerous opportunities to study the sensory ecology of relevant behaviours. Species across the avian family of swallows (Hirundinidae) breed either solitarily or in groups up to thousands of pairs, building their nests in cavities or on hard substrates with overhanging ledges. When foraging in groups, swallows often recruit others to dense food aggregations, serving as information centres. Such food recruitment signals include both specific vocalisations (specific calls) and visual signals (contrasting rump bands potentially visible from a distance and enhanced by the movement of the individual) (Brown et al., 1991). Specifically, contrasting rump bands on the backs of birds have evolved predominantly in colonially nesting and group foraging swallows, whereas the lack of rump bands occurs mostly in solitary lineages (CR Brown, pers. comm.). Such phenotypic adaptations for signalling to conspecifics may also be widespread in other group living and foraging lineages. Turning to another promising context, many seabirds nest in large colonies (across multiple avian orders from pelicans through albatrosses), yet in many lineages indirect cues about the physical locality of the nest provide sufficient sensory identification where dependent offspring predictably occur. In other colonially breeding seabirds, including some penguins and murres, individuals do not build nests and have no permanently identifiable physical structure for where their offspring can be located. Evolutionary theory predicts different acoustic recognition systems and sensitivities in these two ecological contexts: in the latter, for example, paired individual recognition of adult-chick vocalisations is essential for kin-directed behaviours, whereas indirect visual cues suffice for parent–offspring recognition in the former (Lefevre et al., 1998).

Animals living and foraging in crowded group contexts must differentiate prey-sourced stimuli from those of nearby conspecifics. For example, African social spiders (*Stegodyphus dumicola*) live in groups of up to a few hundred individuals, constructing and sharing the same web. Here, prey detection cues need to be distinguished from the large amount of background noise of nest conspecifics (Wright et al., 2020). Experimental work has shown that this species responds more to pulsed vibrational cues that might reflect a prey capture than to continuous vibrational cues that are more likely to reflect conspecific activity (Wright et al., 2020). This scenario seems likely to create considerable selective pressures on sensory systems and sensory input interpretation. Turning to a different sensory system, echolocating bats must deal with acoustic masking—that is, interference caused by conspecific bats emitting calls with similar frequencies that prevents nearby bats from detecting and processing the echoes from their own calls—when hunting in aggregations. Mathematical modelling and simulation approaches have proven to be highly useful in understanding the masking problem, and in developing predictions as to whether bats should intentionally shift their signal frequencies to improve prey capture in crowded situations (Mazar and Yovel, 2020). Such studies employ careful parameterisation of naturalistic stimuli (prey noises, stimuli from self, and conspecifics), in-depth understanding of receiver inputs based on animal sensory filters and amplifiers, and records of natural behaviours and responses under different scenarios to generate novel insights and refined hypotheses.

Even ‘solitary’ species, such as *Drosophila melanogaster* (Gullan and Cranston, 2010), will aggregate in some contexts, which provides useful insights into sensation and collective behaviour (Schneider et al., 2012). In response to an aversive odour, *Drosophila* show a collective avoidance response (Ramdya et al., 2015). This collective odour avoidance arises from cascades of appendage touch interactions between pairs of flies (Ramdya et al., 2015). Larval *Drosophila* also exhibit collective foraging behaviour—engaging in coordinated dives into the food, in order to access deeper and higher quality food (Dombrovski et al., 2017). Both mechanosensation and vision play a role in this process (Dombrovski et al., 2019). *Drosophila* represent an amenable system to study group-level influences on various behaviours because they permit the combined use of neurogenetic tools to dissect the sensory mechanisms underlying the reported group effects. This combined systems-level and neurogenetic approach on the sensory responses of a solitary species could also be used in other systems to study the comparative structural and functional connectivity between these neurons and sensory modalities. Identifying such model species with well-understood sensory systems and existing molecular and experimental toolkits has considerable promise for developing the study of sensory collectives (Box 3).

Box 3. **The promise of shared model systems in the study of sensory collectives**

In most fields of biological study, organically or in a directed manner, a handful of taxa rise to the level of such intense and productive examination, that they become flagship or model species for their respective discipline (Ankeny and Leonelli, 2020). Species spanning disparate branches of the tree of life, including *Caenorhabditis elegans*, *Macaca mulatta*, and *Drosophila melanogaster*, have provided countless insights into anatomy, physiology, behaviour, neurology, and evolution (Katz, 2016). This is no different for collective behaviour. For example, the zebrafish, *Danio rerio* (Box 3—figure 1), has become a useful model for understanding collective phenomena (Shelton et al., 2020) ranging from visually guided shoaling behaviours (Miller and Gerlai, 2012) and chemically guided alarm (substance) collective responses (Speedie and Gerlai, 2008). The species is also a biomedically relevant neurogenomic, ontogenetic, and ethological model species in the laboratory, with advanced molecular and imaging tools available for studying sensory processes in a developmental context (Veldman and Lin, 2008). More recently (Parichy, 2015), the study of wild zebrafish behaviours, including their shoaling, mating system, and oviposition choices, all aspects of their sensory ecology, has come into focus by several research teams already using the species as a laboratory model system. With increasingly advanced molecular, robotic, and holographic techniques employed in the study of group living in captive zebrafish (De Lellis et al., 2020), the time has come to integrate adaptive sensory systems into experimental investigations of collective behaviours both in the lab and in the wild (Shelton et al., 2020).

**Box 3—figure 1.**
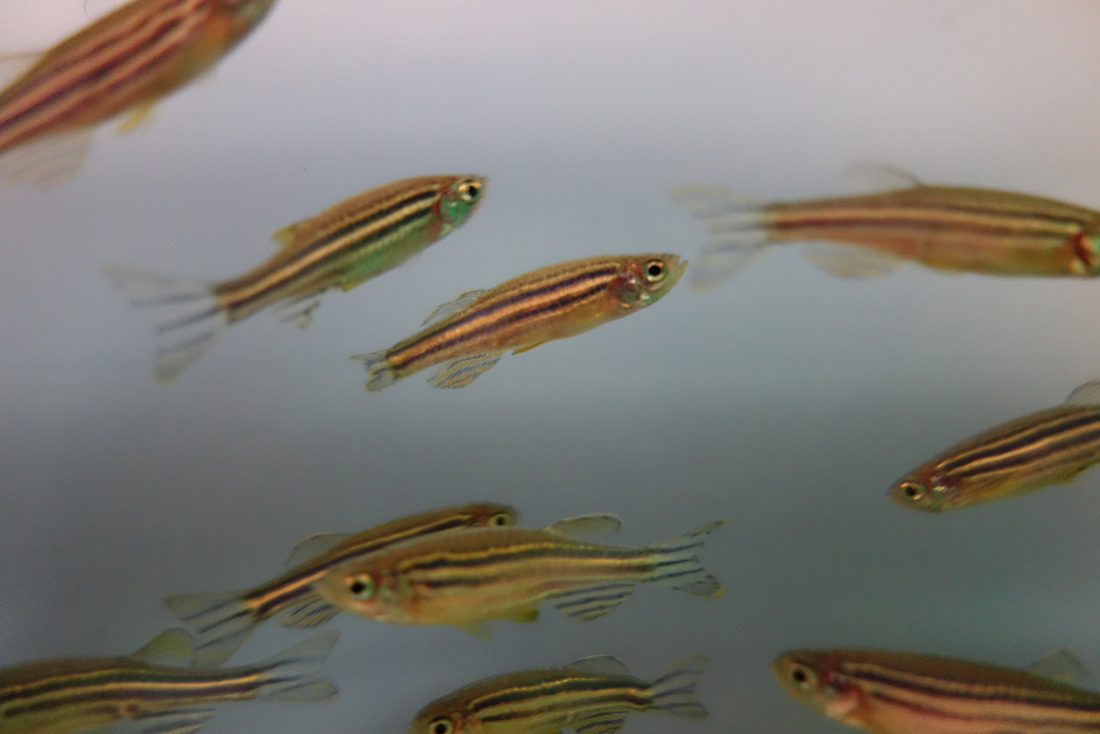
Adult zebrafish always travel in groups, whether in nature or in captive settings.

Photo credit: Delia S. Shelton.

## Conceptual and methodological advances to quantify a ‘sensescape’

A long-standing problem concerns how we may infer stimuli perception from our understanding of the sensory capabilities of individuals and particularly be informed by collective-borne signals and cues, in situ. One approach is to record aspects of the environment that an animal is likely to consider important in their umwelt and infer a sensescape by measuring behavioural response. Although environmental recordings are often limited to extremes in spatio-temporal resolution—the focus being either many measurements across space at low temporal frequency or high-frequency data recorded at a single location or over a narrow temporal window—there are a number of developments that could be made to record the environment at scales reflective of a group’s sensory capacity and perceptual range. These include camera traps or UAVs that can capture the precise environment that an animal or group is exposed to; for example, flying a UAV along the recorded flight path of an aerial species to collect in situ environmental data, equipped with hydrometers, pressure sensors, audio recorders, or miniaturised lidar technology. Advancement in our understanding of the umwelt would require investment in technologies recording the sensescape as part of environmental remote sensing initiatives. Indeed there is already momentum in the remote recording of sound and magnetic data (and in the corresponding development of data standards) for the mapping of sensescapes at global scales but high spatio-temporal resolution (e.g. MagGeo; Benitez-Paez et al., 2021).

Alternatively, we may approach the intersection from the animal’s perspective, that is, how an animal perceives the world around it given its sensory capacity. Some established methods from behavioural ecology that could be combined with methods in sensory ecology include bio-logging (Rutz and Hays, 2009; Williams et al., 2020), virtual reality (Sridhar et al., 2021; Stowers et al., 2017), and robotics (Halloy et al., 2007; Krause et al., 2011). Previous work in collective behaviour has attempted to capture the sensescape by modelling visual perception from position and orientation data of animals and in some cases, combining this with acoustics (Smith and Pinter-Wollman, 2021; Strandburg-Peshkin et al., 2013). While this approach may record sensory inputs directly, we must still consider the perceptual range, including the consideration that the range of the technology may differ from the active space of the animal (Aben et al., 2021) or that noise cannot be partitioned from perceived stimuli. However, some advancements in the sensory capacity of our technologies show huge potential. This includes neurological sensors that may record the processed information of the animal; as seen in examples of 3D-space representation recording in bats (Sarel et al., 2017; Yartsev and Ulanovsky, 2013) and olfactory sensory response recorded with electroencephalogram (EEG) on free roaming animals (Whitford and Klimley, 2019). Further, animal-attached sensors can record aspects of the sensescape that a freely behaving animal is exposed to directly in naturalistic environments, including infrasound recorders onboard seabirds (den Ouden et al., 2021), chemical sensors recording humidity and oxygen saturation (e.g. Wild et al., 2023) which may open the door to further chemical sensors relevant to olfaction (e.g. for mapping odour plume exposure), and use of extreme high-frequency accelerometers to record haptic or vibration by collectives where touch sensation is key (Mariette and Buchanan, 2019; Sumpter et al., 2012). It may also be possible to work with the collective perspective using ‘collective bio-logging’ approaches where animal-attached sensors work together to assimilate information before transmitting their data, essentially operating as a collective to increase the efficiency of data recording and transmission (Wild et al., 2023). Using bio-logging/identification technologies at social sites to record sensory and collective data simultaneously also holds promise (e.g. Ferreira et al., 2020); these new perspectives may allow us to better understand the role of inter-individual differences, group structure, or cohesion in collective behaviour.

Within the realm of collective behaviour, at least two distinct approaches have been used to model the mapping between sensory inputs and behavioural outputs. Classical models in collective behaviour posit a set of simplified behavioural rules (e.g. attraction, alignment, and repulsion) and seek to use those rules to explain behavioural patterns (Couzin et al., 2002). Bringing sensory ecology into this approach could inform how these hypothesised rule-sets should be constructed by highlighting which pieces of sensory information that are likely to be perceived and relevant to any given organism (Kranstauber et al., 2020; Strandburg-Peshkin et al., 2013; Witkowski and Ikegami, 2016). A complementary approach, increasingly used in recent years, is that of machine learning to establish the input–output relationship between high-dimensional sensory inputs and lower-dimensional behavioural outputs, that is, dimensionality reduction (Graving et al., 2019; Graving and Couzin, 2020; Valletta et al., 2017). Within this approach, an understanding of the organism’s sensory capacities would provide a more realistic set of features reflecting the actual perception of the organism, from which the input–output relationship can then be more effectively learned (see Tuia et al., 2022). An additional possible use of this information would be to exclude it from the machine learning algorithm and ask whether the learned relationship uses realistic features, thus shedding light on whether these methods make biological sense.

Once a hypothesised mapping between sensory inputs and behavioural responses has been established, experimental tests are needed to investigate real-life receiver responses and collective action. Manipulations might involve the introduction of artificial cues or signals into a collective system, for example, via audio playback experiments, and presentation of visual or chemical stimuli. The use of robotics in multimodal communication studies has been extensive (Gardner et al., 2021; Patricelli et al., 2007) as it enables individual unimodal components of signals to be presented to receivers in isolation, and then combined with components in other modalities, to investigate the influence of signals both separately and in combination (e.g. Swain et al., 2012). These studies have primarily focused on bimodal signals, and there is a need to increase the number of modalities investigated to more fully describe communication (Higham and Hebets, 2013). However, the power of animal–robot interactions is likely as yet unrealised, in sensory-interactions and information transfer. Only by developing our understanding of the interaction between umwelt and collective behaviour may we advance the use of robotics in collective behaviour studies (Butail et al., 2014; Datteri, 2021; Swain et al., 2012). A further issue in sensory manipulation experiments is that some multimodal signals are ‘fixed’, in that one component of the signal cannot be expressed without the other (e.g. a frog croak that cannot be made without simultaneous inflation of the throat sac), whereas others are ‘free’, with different components of a multimodal display being expressed independently (e.g. visual and vocal components of a bird of paradise display). Depending on the system, it may be easier to present individual unimodal components of a multimodal signal where it is a free signal, than where it is fixed or interconnected.

Another line of potential experimental research involves knocking out or restricting the sensory abilities of individuals within groups, for example, via genetic manipulations affecting the sensory system, physical blocking of an organism’s sensory organs, or the artificial introduction of noise to impede perception of certain sensory channels (Leonard and Horn, 2008; Leonard and Horn, 2005). Such knockouts could either be done globally in a way that affects all group members or locally in a way that targets specific group members (e.g. Ramdya et al., 2015). Manipulations that target only a subset or introduce conflicting information could enable testing of hypotheses regarding the roles that specific individuals play within collective dynamics (Townsend et al., 2012), or how such conflicts are resolved. Finally, studying populations of animals with naturally occurring variation offers opportunities to ask how sensory variation in one system impacts use of other sensory modalities. For example, the widespread colour vision polymorphism found in monkeys in the Americas (Parvorder Platyrrhini) has allowed researchers to ask how dichromatic versus trichromatic colour vision impacts the use of olfaction, taste, and haptic sensation during food investigation within and across species. They have found that dichromatic (red-green colourblind) individuals sniff and bite fruits more often than trichromats investigating the same food items (Melin et al., 2022; Melin et al., 2019).

## Applications to biological conservation

We live in the Anthropocene, an epoch characterised by the dominance of our species. In just over half a century, we have introduced stark changes in the daily and seasonal rhythms of light (Kyba et al., 2023; Kyba et al., 2017), in soundscapes across diverse habitats (Buxton et al., 2017), and in the chemical composition of the air and water bodies that surround us (Lürling and Scheffer, 2007). This sensory pollution has changed the sensescapes experienced by animals, which may exceed their thresholds for plasticity—an inescapable ‘ecological trap’ (Schlaepfer et al., 2002). In many cases, sensory noise pollution has damaged their sensory systems, reduced their active range, and pushed out sensitive species, driving them closer to extinction (Sordello et al., 2020).

Recent work has outlined three main mechanisms by which sensory pollutants impact behavioural and physiological responses in organisms—masking, distracting, and misleading (Dominoni et al., 2020). While this framework provides critical insight into the impact of sensory pollution on organisms, it lacks emphasis on the importance of the social environment in which most species are embedded and the sensory feedback processes that accompany them. We consider each of these mechanisms and highlight testable predictions that emerge from adopting a sensory collective perspective. We also emphasise that these predictions differ from ones that would arise from the original framework where individuals are considered in isolation from their social environment.

Masking occurs when the capacity of an organism to discriminate target stimuli is decreased by the presence of a sensory pollutant with overlapping physical properties. For example, high levels of artificial light at night can mask important cues from moonlight and starlight (Chan et al., 2010; Kyba et al., 2023; Morris-Drake et al., 2016; Storms et al., 2022). Distracting refers to conditions where the sensory pollutant interferes with information processing by occupying the individual’s attention capacity. Distracting stimuli are often associated with diminished learning, spatial orientation, and memory retrieval. For example, high noise levels can distract animals during foraging and vigilance behaviours. In scenarios with masking or distracting pollutants, the integration of noisy signals detected by a fraction of individuals in a sensory collective may, nevertheless, be sufficient to facilitate detection of the target stimulus compared to solitary individuals. Specifically in the context of spatial orientation, models of collective behaviour have shown that a few highly informed individuals or many individuals with noisy information can lead groups to target locations (Couzin et al., 2011; Couzin et al., 2005; Ioannou et al., 2015). Thus, collective dynamics may have some hope of counteracting the negative effects of anthropogenically driven masking and distracting stimuli. Misleading occurs when the sensory pollutant is detected as a natural cue or signal and invokes inappropriate behaviours from organisms. Unlike pollutants that affect organisms via masking or distracting, inappropriate behaviour triggered by sensory pollutants that mislead may be amplified, contributing to the phenomenon of ‘collective stupidity’ and hence resulting in worse outcomes relative to those predicted for isolated individuals. Naturally, the effects of sensory pollutants will strongly depend on the species, modalities, and behaviours involved. A sensory collective perspective on animal responses to sensory pollution will facilitate establishing which species are most impacted by sensory pollution and the social mechanisms in place that may mitigate or exacerbate this impact.

Determining the umwelten of a sensory collective is essential for understanding how animals respond to sensory pollution and anthropogenic change. For example, mass migrations of sea turtles towards artificial lights (Thums et al., 2016) or the collective beaching of pilot whales (Fehring and Wells, 1976) may stem from the social amplification of misinformation at the individual level. Thus, an understanding of sensory biology and sensory pollutants, and the ways sensory information is integrated by the collective, is important for developing effective wildlife management and conservation policies.

## Closing remarks

More than 100 years ago, the biologist Jakob von Uexküll argued that animals inhabit different sensory worlds (umwelten) even while occupying the same environment. This insight informs the fields of neuroethology and sensory ecology, but it is rarely addressed in the emerging field of collective behaviour, which draws on the collective and emergent properties of animal groups. The goal here was to bridge these topics and advance their respective areas of enquiry. Both areas have burgeoned in recent years, although to date they have often done so quite separately. An improved understanding of what collective umwelten might look like has the potential to provide new insight into the emergent properties of collectives, while improving our understanding of the suite of selective pressures influencing the evolution of sensory systems. Our synthesis has also highlighted numerous areas that are likely to prove fruitful for further investigation. There are major outstanding questions surrounding the extent to which the generation of information in many individuals via sensory stimulation, followed by the collective sharing of that information via communication, is likely to create increased noise versus informational enhancement across the collective, which could either cloud or improve the effectiveness of individual decision-making. Given the increasing encroachment of human impacts on animal populations, improving our understanding of how anthropogenic impacts, such as light and noise pollution, interfere with sensescapes and hence might interfere with collective animal movements and actions seems imperative.
